# Immune Checkpoint Inhibitors in the Management of Urothelial Carcinoma

**DOI:** 10.33696/cancerimmunol.3.047

**Published:** 2021

**Authors:** Aakash Patel, Daniel I. Bisno, Hiren V. Patel, Saum Ghodoussipour, Biren Saraiya, Tina Mayer, Eric A. Singer

**Affiliations:** 1 Rutgers Robert Wood Johnson Medical School, New Brunswick, NJ 08901, USA; 2 Section of Urologic Oncology, Rutgers Cancer Institute of New Jersey, New Brunswick, NJ 08903, USA; 3 Division of Medical Oncology, Rutgers Cancer Institute of New Jersey, New Brunswick, NJ 08903, USA

**Keywords:** Urothelial carcinoma (UC), Bladder cancer, Upper tract urothelial carcinoma (UTUC), Immunotherapy, Immune checkpoint inhibitors, PD-1, PD-L1

## Abstract

Urothelial carcinoma is one of the most common cancers in the United States, yet outcomes are historically suboptimal. Since 2016, the approval of five programmed cell death 1 and programmed death-ligand 1 immune checkpoint inhibitors for locally advanced and metastatic urothelial carcinoma has led to improved oncologic outcomes for many patients in the second-line setting. Two checkpoint inhibitors, pembrolizumab and atezolizumab subsequently earned approval for first-fine therapy with restricted indications. More recently, pembrolizumab was approved for bacillus Calmette-Guérin-unresponsive high-risk non-muscle invasive bladder cancer, opening the door for other immune checkpoint inhibitors to be integrated into treatment in earlier disease stages. Recent bacillus Calmette-Guérin shortages have highlighted the need for alternative treatment options for patients with non-muscle invasive bladder cancer. Currently, there are no FDA-approved checkpoint inhibitors for non-metastatic muscle-invasive bladder cancer. Furthermore, many patients are ineligible for standard cisplatin-based chemotherapy regimens. Numerous ongoing clinical trials are employing immune checkpoint inhibitors for muscle-invasive bladder cancer patients in the neoadjuvant, adjuvant, perioperative, and bladder-sparing setting. Although up to 10% of urothelial carcinoma tumors arise in the upper urinary tract, few studies are designed for this population. We highlight the need for more trials designed for patients with upper tract disease. Overall, there are numerous clinical trials investigating the safety and efficacy of immune checkpoint inhibitors in all stages of disease as single-agents and combined with dual-immune checkpoint inhibition, chemotherapy, radiotherapy, and other pharmacologic agents. As the field continues to evolve rapidly, we aim to provide an overview of recent and ongoing immunotherapy clinical trials in urothelial carcinoma.

## Introduction

Bladder cancer is one of the most common and expensive cancers in the United States, with an expected 81,400 new cases and 17,980 deaths in 2020 alone [1–3]. The incidence is increased among white men and diagnoses often occur in the 7^th^ decade of life [4–6]. The most common type of bladder cancer is urothelial carcinoma (UC), formerly referred to as transitional cell carcinoma. Less than 10% of cases of UC originate in the upper urinary tract, which includes the renal calyces, renal pelvis, and ureters [7–9]. Common risk factors for upper tract UC (UTUC) include smoking and occupational exposures, as well as hereditary nonpolyposis colorectal syndrome (HNPCC or Lynch syndrome) and dietary intake of aristolochic acid [10,11].

Since 2016, the U.S. Food & Drug Administration (FDA) has approved multiple agents targeting the immune pathway. Programmed cell death 1 (PD-1) is a receptor expressed on host immune cells [12]. Tumor cells can downregulate the immune response by expressing programmed death-ligand 1 (PD-L1), which leads to the inhibition of cytokine release and T-cell clonal expansion [13,14]. Inhibiting this pathway with antibodies targeting PD-1 and PD-L1 has demonstrated activation of robust antitumor responses against several solid tumors, including UC. Given the success of these immune checkpoint inhibitors (ICIs) in metastatic UC (mUC), there has been a growing interest in incorporating checkpoint blockade into earlier stages of UC. Researchers are also investigating anti-PD-1/PD-L1 ICIs in combination with anti-cytotoxic T-lymphocyte-associated antigen-4 (CLTA-4) ICIs, various chemotherapy regimens, and radiotherapy regimens to enhance therapeutic strategies. In this review, we discuss notable recent and ongoing phase 2 and 3 immunotherapy clinical trials in a) mUC, b) muscle-invasive bladder cancer (MIBC), and c) non-muscle invasive bladder cancer (NMIBC).

## First-line Immunotherapy in Metastatic UC

Preferred first-line treatment of mUC in cisplatin-eligible patients includes chemotherapy with gemcitabine and cisplatin or dose-dense methotrexate, vinblastine, doxorubicin, and cisplatin (ddMVAC) [15–17]. For cisplatin-ineligible patients, pembrolizumab and atezolizumab are currently FDA-approved as first-line agents for those with tumors with high PD-L1 expression, or who are ineligible for all platinum-based chemotherapies regardless of PD-L1 expression [18].

Accelerated approval of pembrolizumab for first-line mUC treatment was a result of KEYNOTE-052, a single-arm, phase II study of cisplatin-ineligible patients with mUC who were administered intravenous pembrolizumab every three weeks. A favorable objective response rate (ORR) and an even greater response in high PD-L1 patients led to accelerated FDA approval. Updated long-term outcomes are shown in Table 1. Approximately 20% of patients had treatment-related adverse events of grade 3 or greater, most commonly fatigue and colitis [19]. With these encouraging results, a subsequent phase III trial, KEYNOTE-361, was undertaken to compare pembrolizumab with or without chemotherapy to chemotherapy alone. Preliminary data demonstrated reduced survival among low PD-L1 expressors on pembrolizumab monotherapy compared to those on chemotherapy. Due to this, the FDA revised the initial indication for first-line pembrolizumab to include a requirement for high PD-L1 expression for cisplatin-ineligible patients [18]. Recently, investigators announced that the study did not meet its primary endpoints of a statistically significant improvement in overall survival (OS) and progression free survival (PFS) in the combination group relative to chemotherapy alone [20]. As this data is pending presentation, interpretation of these findings should be limited.

Results from phase II and phase III trials examining the use of atezolizumab as a first-line agent have been encouraging. IMvigor210 (Cohort 1) is a single-arm, phase II study of cisplatin-ineligible patients with mUC given intravenous atezolizumab every 21 days. As shown in Table 1, both IMvigor210 and KEYNOTE-052 show similar ORR for all patients and respectively higher ORR for those with high PD-L1 expression [21]. Similar to KEYNOTE-052, original IMvigor210 Cohort 1 data show that 16% of patients experienced grade 3 or 4 treatment-related adverse events including fatigue and transaminitis [22]. Preliminary data from IMvigor130, a three-arm phase III trial comparing atezolizumab with or without chemotherapy to chemotherapy alone, has also demonstrated a survival reduction among low PD-L1 expressors on atezolizumab monotherapy relative to chemotherapy. This led to a similar restriction for atezolizumab monotherapy, as was previously noted for pembrolizumab monotherapy, to only high PD-L1 expressors. Recently published IMvigor130 results demonstrated prolonged PFS with combination atezolizumab and chemotherapy of 8.2 months versus 6.3 months with chemotherapy alone. This prolongation of PFS is unique to atezolizumab, as KEYNOTE-361 did not show prolonged PFS for pembrolizumab per trial investigator announcement, as previously discussed [20,23]. While a PFS prolongation of approximately 2 months may be seen as providing marginal benefit, combination of atezolizumab and chemotherapy also showed nearly twice the complete response rate relative to chemotherapy alone (13% versus 7%, respectively) with similar safety profiles. With these encouraging results, clinicians should closely examine ongoing atezolizumab trial data for treatment consideration in appropriate patient populations.

There are many ongoing studies for first-line immunotherapy in mUC as shown in Table 2. This includes two phase III studies: LEAP-011 and NILE. LEAP-011 is investigating pembrolizumab and lenvatinib, a multiple tyrosine kinase inhibitor (TKI) targeting vascular endothelial growth factor receptor (VEGFR), fibroblast growth factor receptor (FGFR), and platelet-derived growth factor receptor (PDGFR) [24]. In contrast, NILE includes two ICIs, durvalumab and tremelimumab, an anti-CTLA-4 antibody [25]. Phase II studies for first-line immunotherapy currently include the agents that have already gained FDA approval as second-line treatments, namely nivolumab, avelumab, and durvalumab. There are also multiple phase II studies of combination treatments, which are discussed later. This rapidly advancing field warrants frequent updates on trials and their results.

## Second-line and Subsequent-line Immunotherapy in Metastatic UC

ICIs were first approved in mUC as second-line therapies in the post-platinum setting (Table 3). Atezolizumab was approved due to IMvigor210 (Cohort 2), a phase II trial of atezolizumab in progressed mUC that demonstrated an improved ORR relative to a historical ORR of 10% for second-line chemotherapy and a marked improvement in ORR and median OS among high PD-L1 expressors [21,26]. Despite these positive findings, a phase III trial, IMvigor211, did not show a statistically significant improvement in median OS relative to chemotherapy [27]. However, atezolizumab treatment led to fewer treatment-related adverse events relative to chemotherapy at 20% versus 43%, respectively. Following the results of IMvigor211, the second-line indication for atezolizumab was withdrawn in March 2021 [28]. IMvigor130, which studies atezolizumab in the first-line setting, will continue until final analysis. Second-line pembrolizumab approval followed KEYNOTE-045, a phase III trial comparing pembrolizumab with chemotherapy consisting of paclitaxel, docetaxel, or vinflunine [29, 30]. The pembrolizumab group not only had greater OS, but also fewer treatment-related adverse events of 62% versus 90.6% in the chemotherapy group.

While only pembrolizumab and atezolizumab are currently FDA approved as both first- and second-line agents, three other ICIs are approved as second-line agents: nivolumab, durvalumab, and avelumab (Table 3). Approval for nivolumab was based on CheckMate275, a single-arm phase II study of nivolumab that demonstrated an ORR of 19.6% (95% CI 15.0-24.9%), similar to the ORR in IMvigor210 of 16% (95% CI 13-21%). Comparable to IMvigor210, CheckMate275 demonstrated grade 3 to 4 treatment-related adverse events in 18% of patients, most commonly fatigue and diarrhea [31]. Durvalumab was FDA approved based on a phase I/II study with a similar ORR to other second-line ICIs [32]. Grade 3 to 4 treatment-related adverse events occurred in 6.8% of patients. Subsequently, the results of DANUBE, a first-line phase III trial, did not reach its coprimary endpoints of OS in patients treated with combined durvalumab plus tremelimumab compared to chemotherapy and OS in high PD-L1 expressors who received durvalumab alone compared to chemotherapy [33]. In February 2021, the second-line indication for durvalumab was withdrawn [34]. Avelumab was approved based on a phase I trial with pooled results from two cohorts of patients in the JAVELIN Solid Tumor study, which displayed a similar ORR to the above ICIs. Grade 3 to 4 treatment-related adverse events occurred in 8% of patients [35]. Recently reported, in patients without progression after four to six cycles of platinum based chemotherapy, avelumab maintenance therapy has been found to improve OS compared to supportive care based on an interim analysis of JAVELIN Bladder 100, an ongoing phase III study of avelumab with supportive care versus supportive care alone [36]. Median OS on avelumab was 21.4 months versus 14.3 months on supportive care (HR 0.69, 95% CI 0.56 to 0.86, 1-sided p=0.0005). Among high PD-L1 expressors, the median OS was not reached on avelumab versus 17.1 months on supportive care (HR 0.56, 95% CI 0.40 to 0.79, 1-sided p=0.0003). Grade 3 or greater adverse events occurred in 47.4% of avelumab patients versus 25.2% of supportive care treated patients. Based on these results, avelumab has also been FDA approved for post-platinum maintenance therapy in mUC. In a phase II study, NCT02500121, comparing maintenance pembrolizumab and placebo in patients with mUC demonstrating at least stable disease on platinum-based first-line chemotherapy, pembrolizumab showed a greater objective response (23% versus 10%), PFS (5.4 months versus 3.0 months), and median OS (22 months versus 18.7 months) compared to placebo, further supporting the role of maintenance immunotherapy in mUC [37].

With multiple FDA approved second-line ICIs, ongoing trials with ICI combination regimens are underway as shown in Table 4. One phase III study, NCT03390504, is included in this group as one treatment arm is given pembrolizumab [38]. However, the focus of this study is erdafitinib, a FGFR inhibitor. Similar to this study and given the rapid expansion of this field, many of the second-line trials focus on experimental medications in combination with ICIs, as discussed as follows.

## Combination Immunotherapy in Metastatic UC

Many combinatorial studies are investigating ICIs with growth factor inhibitors, such as PemCab, a first-line, phase II, single group study combining pembrolizumab and cabozantinib, a multiple TKI [39]. Other first-line studies are also examining ICIs in combination with growth factor inhibitors, such as LEAP-011 with lenvatinib, NCT03272217 with vascular endothelial growth factor (VEGF) inhibitor bevacizumab, and NCT03473756 with FGFR inhibitor rogaratinib [24,40,41]. Second-line studies with ICI and growth factor inhibitor combinations are listed in Table 4. One recently published second-line, phase II study, RAPID CHECK, comparing combination pembrolizumab and acalabrutinib, a Bruton’s tyrosine kinase (BTK) inhibitor, versus pembrolizumab alone in platinum resistant mUC found no significant improvement in OS, PFS, or ORR with combination therapy and also showed higher adverse event rates. Despite these results, combination therapy resulted in increased CD8^+^ T-cell proliferation [42]. Further studies will be needed to determine if this immune stimulation results in increased tumor infiltration. Overall, the goal of these studies is to determine if targeting two different mechanisms of oncogenesis can provide a benefit over targeting these mechanisms in exclusion.

Combination studies are also seen with two ICIs together as well, all of which currently feature a PD-1/PD-L1 inhibitor combined with a CTLA-4 inhibitor. First-line studies include NCT03682068 (NILE) with durvalumab and tremelimumab, and NCT03036098 (CheckMate901) with nivolumab and ipilimumab [25,43]. CheckMate901 follows encouraging results from a phase I/II post-platinum trial of nivolumab with ipilimumab that demonstrated a greater ORR of 26.9% and 38.0% in the combination groups of two different dosage regimens relative to an ORR of 25.6% with nivolumab alone, while also maintaining comparable safety profiles [44]. One second-line study NCT03871036 (ICRA) includes durvalumab and tremelimumab [45].

Expansion beyond these treatment classes is also occurring rapidly, with novel medications of different mechanisms of action being examined with ICIs. First-line studies include NCT03288545 (EV-103), NCT03459846 (BAYOU), and NCT03785925 (PIVOT-10) [46–48]. Encouraging preliminary results were recently presented for EV-103 which is investigating first-line enfortumab vedotin, an antibody-drug conjugate targeting a cancer-associated cell surface protein nectin-4, with pembrolizumab in cisplatin-ineligible mUC. EV-103 demonstrated an ORR of 73.3% (95% CI 58.1-85.4%) overall and an ORR of 78.6% in high PD-L1 expressors [46]. Second-line studies in this category are listed in Table 4. As this field continues to grow, further unique mechanisms of oncogenic inhibition will continue to be explored.

Despite advances in the field, not all combination therapies result in a favorable outcome. KEYNOTE-672 investigated pembrolizumab monotherapy versus pembrolizumab with epacadostat, an indoleamine 2,3-deioxygenase 1 (IDO1) inhibitor [49]. While preliminary data shows an improved ORR with combination therapy of 31.8% (95% CI 22.46 to 55.24%) versus 24.5% (95% CI 15.33 to 43.67%) with pembrolizumab monotherapy, there is a higher all-cause mortality of 29.55% versus 20.41% respectively. As these preliminary results have not yet been discussed in a peer reviewed publication, interpretation of these findings should be limited. Concurrent with these preliminary results is KEYNOTE-252, which investigates the same drug combination but in metastatic melanoma, and showed no improvement in PFS or OS when compared to pembrolizumab alone [50]. Thus, novel therapies must be carefully considered for inclusion in upcoming trials.

## Muscle-Invasive Bladder Urothelial Carcinoma

Patients diagnosed with non-metastatic MIBC (pT2 or greater) are initially assessed for surgical candidacy. Candidates for surgery are recommended to undergo neoadjuvant chemotherapy followed by radical cystectomy and pelvic lymph node dissection [51–53]. Cisplatin is the cornerstone of neoadjuvant chemotherapy [52]. However, up to 50% of UC patients are cisplatin-ineligible due to renal insufficiency, peripheral neuropathy, and other comorbidities [54]. Patients who are at significant risk of disease progression are considered for concurrent chemoradiation therapy [55]. Taken together, this underscores the importance of alternative treatments options for MIBC [54,56,57].

Given the success of neoadjuvant cisplatin in MIBC, many ICI studies utilize cisplatin in the investigational drug regimen. Numerous trials combine gemcitabine-cisplatin with ICIs such as nivolumab (BLASST-1 NCT03294304), pembrolizumab (NCT02690558, HCRN GU14-188 NCT02365766), avelumab (AURA NCT03674424), and investigational PD-1 antibody toripalimab (NCT04099589) [58–60]. BLASST-1 results report positive safety and efficacy data, including 66% pathologic downstaging (≤ pT1No) [61]. In HCRN GU14-188, pembrolizumab and gemcitabine-cisplatin led to a 61.1% pathologic downstaging and 44.4% pathologic complete response (pCR) rate (pTo) in cisplatin-eligible patients [62].

Despite neoadjuvant cisplatin being the gold standard, problems with eligibility due to risks of this regimen have led to numerous trials designed to address the cisplatin-ineligible patient population. Neoadjuvant atezolizumab (ABACUS, NCT02662309, NCT02451423) and pembrolizumab (PANDORE, NCT03212651) are being studied in single-arm phase 2 trials. In the ABACUS trial, patients received two courses of atezolizumab prior to radical cystectomy resulting in a 31% pCR rate [63]. HCRN GU14-188 included a cisplatin-ineligible arm that received pembrolizumab and gemcitabine [64]. Interim results show 51.6% pathologic downstaging and 45.2% pCR rate. In both arms of HCRN GU14-188, response rates did not correlate with PD-L1 scores.

Neoadjuvant pembrolizumab monotherapy was recently studied in multiple single-arm phase 2 studies (PURE-01, NCT03319745). In PURE-01, patients received three courses of pembrolizumab prior to radical cystectomy [65,66]. There was a 39% pCR rate and 56% pathologic downstaging, further supporting the efficacy of ICIs in the neoadjuvant setting. The surgical safety of radical cystectomy and pelvic lymph node dissection following the administration of pembrolizumab was also shown in a separate report where there were no perioperative mortalities at 90 days and 34% of patients experienced high-grade (≥ 3a) complications, which is comparable to radical cystectomy following chemotherapy [67]. Both ABACUS and PURE-01 (NCT02736266) report promising analyses with candidate biomarkers. However, randomized controlled trials are needed to strengthen findings from these studies [63,68].

Ongoing neoadjuvant approaches employ a combination of ICIs. Durvalumab is being studied in combination with anti-CTLA-4 antibody tremelimumab (DUTRENEO NCT03472274), nivolumab with anti-CTLA-4 antibody ipilimumab (NCT03520491), and nivolumab with CD137 agonist antibody urelumab (NCT02845323) [69]. In DUTRENEO, patients with tumors that had a high pro-inflammatory interferon-gamma signature (tumor immune score, TIS) were randomized to durvalumab with tremelimumab versus chemotherapy, and patients with low TIS tumors received chemotherapy, resulting in pCR rates of 34.8%, 36.4%, and 68.8%, respectively). Although TIS failed to predict response, patients with high PD-L1 expression showed a greater response compared to patients with low PD-L1 expression (pCR = 57.1% and 14.3%, respectively) [70]. NEMIO is a phase 1/2 study investigating durvalumab and ddMVAC with or without tremelimumab (NCT03549715) [71]. Other studies combine neoadjuvant ICI with investigational or off-label agents, including pembrolizumab with investigational entinostat (NCT03978624), a selective class I histone deacetylase inhibitor, and epacadostat (PECULIAR NCT03832673); and atezolizumab with cabozantinib (NCT04289779), a small molecule TKI [72]. Promising preliminary data offers the possibility of expanding the neoadjuvant repertoire for MIBC.

Data supporting the use of adjuvant immunotherapy is limited, however some studies have shown benefit [73,74]. There are multiple randomized phase 3 studies comparing adjuvant ICI use to observation in MIBC at high-risk for recurrence using atezolizumab (IMvigor010 NCT02450331), pembrolizumab (AMBASSADOR NCT03244384), and nivolumab (CheckMate 274 NCT02632409), as well as a randomized phase 2 study in MIBC using adjuvant durvalumab (NCT03768570) [75]. These studies include some patients who have had chemotherapy prior to surgery. IMvigor010 did not reach its primary endpoint of disease-free survival (DFS) for patients treated with adjuvant atezolizumab compared to control patients (median DFS = 19.4 months and 16.6 months, respectively). Furthermore, there are multiple randomized phase 3 studies employing ICI use perioperatively, both before and after radical cystectomy, using pembrolizumab (KEYNOTE-905 NCT03924895), pembrolizumab with gemcitabine-cisplatin (KEYNOTE-866, NCT03924856), and durvalumab with gemcitabine-cisplatin (NIAGARA NCT03732677), nivolumab with bempegaldesleukin (NCT04209114), an investigational CD122-preferential IL-2 pathway agonist, and nivolumab with linrodostat, an ID01 inhibitor, and chemotherapy (ENERGIZE NCT03661320) [76–79].

Given the number of patients who are ineligible or who chose not to undergo surgery, there is an ongoing interest in bladder-sparing approaches. CRIMI is a phase 1/2 study investigating nivolumab and ipilimumab with mitomycin, capecitabine, and radiotherapy in two weight-based dosing arms versus fixed dose nivolumab with chemoradiotherapy (NCT03844256). Ongoing phase 2 studies include durvalumab with tremelimumab and radiotherapy (IMMUNOPRESERVE NCT03702179), nivolumab with gemcitabine-cisplatin (NCT03558087), and atezolizumab with radiotherapy (NCT04186013) [80]. Details of phase 2 and 3 bladder-sparing studies employing ICI with chemoradiotherapy are in Table 5 [80–84].

## Non-Muscle Invasive Bladder Urothelial Carcinoma

Non-muscle invasive bladder cancer (NMIBC; Ta, T1, and Tis) is treated with transurethral resection of the bladder tumor (TURBT) and intravesical chemotherapy or immunotherapy [15,85]. In patients with intermediate or high-risk NMIBC, intravesical Bacillus Calmette-Guérin (BCG) can be used as local immunotherapy [86,87]. However, after BCG therapy, as many as 80% of NMIBC patients will experience disease recurrence and up to 45% will have disease progression [88].

The success of checkpoint blockade in mUC has led to the development of numerous studies incorporating ICIs in the treatment of BCG-refractory high-risk NMIBC. Pembrolizumab was recently investigated in the single-arm phase 2 KEYNOTE-057 study for patients who were unfit or unwilling to undergo radical cystectomy (NCT02625961) [89,90]. Patients received pembrolizumab every 3 weeks for up to 24 months or until unacceptable toxicity, persistent or recurrent high-risk NMIBC, or progressive disease. The complete response (CR) rate was 41% at 3 months and the median duration of response in responders was 16.2 months. Pembrolizumab was discontinued in 11% of patients, most commonly due to pneumonitis. On January 8, 2020, the FDA approved pembrolizumab for the treatment of patients with BCG-refractory high-risk NMIBC with *carcinoma in situ* (CIS) with or without papillary tumors who are unfit/unwilling to undergo cystectomy [91]. Similar phase 2 studies with atezolizumab (SWOG S1605 NCT02844816, NCT02451423), durvalumab (NCT02901548) are currently ongoing [92]. SWOG 1605 focused on a subset of patients with CIS showing that 41% and 26% of patients achieved complete remission at 3 and 6 months, respectively [93]. NCT02451423 employs sequentially increasing dose-level cohorts by enrollment. Although ICIs are typically administered intravenously, durvalumab is being investigated with intravesical administration in a phase 2 study to minimize systemic toxicity (NCT03759496).

Several studies are employing multi-therapeutic approaches with ICIs in the setting of BCG-refractory high-risk NMIBC. Several ICIs are being investigated in combination with BCG, chemotherapy, or radiotherapy in phase I/II, II, and III studies described in Table 6 [94,95]. Nivolumab is being studied with or without linrodostat, and with or without BCG in a randomized phase 2 study (CheckMate 9UT NCT03519256) [96,97]. Pembrolizumab is being studied with CG0070, an oncolytic serotype-5 adenovirus, in a single-arm phase 2 study (NCT04387461). Durvalumab is being studied with S-488210/S-488211, a 5-peptide cancer vaccine, in a single-arm phase 1/2 study (DURANCE NCT04106115).

Given recent reports of shortages of BCG availability in the USA, many researchers are interested in using ICIs in the first-line setting for high-risk NMIBC [98–101]. ALBAN is a phase 3 randomized trial comparing atezolizumab with BCG and BCG monotherapy in BCG-naive patients (NCT03799835) [102]. Similarly, POTOMAC is a phase 3 randomized trial comparing durvalumab with BCG induction/maintenance dual-therapy, durvalumab with BCG induction dual-therapy, and BCG induction/maintenance dual-therapy in BCG-naive patients (NCT03528694) [103]. Pembrolizumab monotherapy is being studied in a single-arm phase 2 study in BCG-naive patients (NCT03504163). Details of NMIBC trials are summarized in Table 6.

## Upper-tract Urothelial Carcinoma

UTUC exhibits a higher incidence of invasive disease at the time of diagnosis relative to UC of the bladder [9]. Therefore, UTUC is often treated with nephroureterectomy and adjuvant chemotherapy [104]. For low risk UTUC, nephron-sparing surgery may be considered, while metastatic disease is treated with systemic chemotherapy [51].

There are few ongoing immuno-oncology trials designed for UTUC patients alone. In one single-arm phase 2 study, patients with high-risk UTUC (CIS, Ta, T1) who are unfit or unwilling to undergo a nephroureterectomy receive pembrolizumab and BCG after endoscopic ablation (NCT03345134) [105]. UTUC patients are often permitted to enroll in UC studies where the majority of patients have tumor originating in the bladder. For instance, in IMvigor010, 13% of patients had UTUC, however the results were not reported by disease site [106]. In KEYNOTE-052, 69 out of 370 patients had a primary tumor location in the upper urinary tract. The ORR was 26.1% and 29.3% for UTUC and lower-tract, respectively, and the median OS was 10.8 months and 11.5 months, respectively [19]. In IMvigor210, 33 out of 119 patients had a primary tumor location in the upper urinary tract. The ORR was 39% and 17% for UTUC and lower-tract, respectively [22]. These data support the clinical efficacy of first-line pembrolizumab or atezolizumab for cisplatin-ineligible locally advanced or metastatic UTUC patients, however further clinical trials are needed.

Regarding second-line ICI therapy in metastatic UTUC patients, limited subgroup analyses have been performed in some of the previously discussed studies. In IMvigor211, a subgroup analysis of 234 high PD-L1 expressors demonstrated that 51 of these patients had UTUC. Among UTUC patients compared to all high PD-L1 expressors, there was a trend towards a less favorable HR for death, although not statistically significant, with atezolizumab treatment relative to chemotherapy at a HR of 0.81 (95% CI 0.59-1.10) among all high PD-L1 expressors, a HR of 1-32 (95% CI 0.50-3.48) among renal primary patients, and a HR of 0.92 (95% CI 0.36-2.34) among ureter primary patients [27]. KEYNOTE-045 supplementary materials note that 38 patients (14.1%) had upper tract primary tumor sites but does not include a subgroup analysis of these patients [29]. CheckMate275 does not specify the proportion of patients with upper tract disease and NCT01693562 does not specify if upper tract patients are included [31,32]. JAVELIN data for avelumab includes a subgroup analysis of 36 upper tract patients out of 161 total patients, and notes a poorer ORR among upper tract patients of 11% versus an ORR of 18% among lower tract patients [35]. Together, these limited findings suggest that patients with primary upper tract disease may have less favorable responses to second-line ICI therapy than lower tract patients and demonstrates the need for further upper tract-specific studies. Metastatic UC trials, as listed in Tables 1–4, either explicitly state that UTUC patients are enrolled, or their enrollment is inferred in studies that do not differentiate by UC site.

The small UTUC sample sizes in these studies likely limit the power for UTUC-specific analyses. This is both a consequence of the lower incidence of UTUC relative to UC originating in the bladder and trial designs, which exclude UTUC patients from enrolling. UTUC-specific studies are an area in need of further contribution.

## Immunotherapy Biomarkers in Urothelial Carcinoma

Given the varied response rate to ICIs based on cellular markers, further studies into biomarkers are warranted in the UC population. Improvements in this respect will benefit trial design, treatment selection, and patient counseling. PD-L1 expression is the most frequently used biomarker in clinical trial designs studying ICIs in UC treatment. PD-L1 expression is currently calculated by two different methods. A combined positive score (CPS), which is the percentage of PD-L1 positive cells in a tumor sample, of greater than 10 represents high PD-L1 expression [107]. The second criteria for high PD-L1 expression is defined as having a tumor sample with 5% or greater of tumor-infiltrating immune cells (IC) stain positive for PD-L1 [22]. The FDA has approved multiple diagnostic tests to measure PD-L1 expression [108,109]. In a recent meta-analysis of 9 clinical trials comprising 1,436 patients, patients with high PD-L1 expression had significant improvements in ORR relative to low PD-L1 expression. PD-L1 expression was better at predicting ORR for patients treated with atezolizumab, durvalumab, and pembrolizumab, compared to nivolumab and avelumab[110]. Further, PD-L1 expression predicted one-year OS for patients treated with PD-L1 inhibitors, but not PD-1 inhibitors. Further description of the relationship between PD-L1 expression and oncologic outcomes is described above for select trials.

In addition to using PD-L1 expression as a biomarker for ICI therapy responsiveness, other biomarkers such as tumor mutational burden (TMB), DNA damage response (DDR) gene defects, and microsatellite instability (MSI) are being studied as markers to predict susceptibility to ICI therapy. TMB refers to the mutation count per coding region in the genome. IMvigor210 performed a subgroup analysis with TMB and found correlations with both greater response rates and longer OS in patients with higher TMB [111]. One study performed genetic sequencing of patients with ICI treated non-small cell lung cancer and found that TMB and PD-L1 expression are not correlated and are both comparable in predicting responsiveness to ICIs [112]. Further TMB studies are warranted to determine if TMB can serve as an independent and validated biomarker for ICI responsiveness in UC.

Defects in DDR genes have been associated with TMB and are also being investigated for predicting ICI responsiveness. In a study of 60 mUC patients enrolled in various ICI treatment trials, DDR gene deletions and high TMB were both associated with greater response rates and OS. Concordant with the known association between DDR gene defects and TMB, this study found that these biomarkers are not mutually independent. When performing multivariable analyses, DDR defect status was found to be superior to TMB at predicting ICI response based on regression modelling goodness of fit [113]. Thus, DDR defect status should also be investigated concurrently with TMB as a biomarker for ICI responsiveness.

Microsatellite instability, characterized by DNA mismatch repair deficiencies, has also been correlated with TMB and ICI response in UC [114]. Of particular importance given the need for dedicated UTUC analysis, one recent study of 128 UTUC patients found that 28.1% of patients demonstrated MSI [115]. With pembrolizumab FDA approval for progressed metastatic high MSI solid tumors and recent approval for first-line treatment of high MSI metastatic colorectal cancer, further investigation into MSI as a biomarker for UC, and particularly UTUC, should be performed [116,117].

## Immune-Related Adverse Events

Given the role that ICIs play in potentiating the immune response to tumor antigens through inhibiting self-tolerance, ICIs may also activate the immune response against self-antigens in healthy tissues outside of the tumor microenvironment leading to numerous inflammatory toxicities known as immune-related adverse events (irAEs). These ICI side effects can often substantially differ from cytotoxic chemotherapy side effects. irAEs can potentially affect any organ. The prevalence of irAEs is up to 70% and 90% in patients treated with a PD-1/PD-L1 inhibitor and CTLA-4 inhibitor, respectively, with mild to moderate skin and gastrointestinal irAEs being more common and the rates of grade 3 and 4 toxicities being fairly low [118,119]. Table 7 summarizes irAEs documented during the use of ICIs [120]. Clinical Practice Guidelines published recently by the American Society of Clinical Oncology recommends continuing therapy with close-monitoring for most grade 1 toxicities, whereas subsequently higher grade toxicities may call for suspension of the ICI and in some cases the use of corticosteroids, immunosuppressive therapy (e.g., infliximab), or other interventions [120]. Most patients tolerate immune therapy, and patients with mild side effects often continue therapy with minimal impact on quality of life. The decision to continue therapy or resume therapy following cessation due to irAEs maybe influenced by other factors including the patient’s tumor response or biomarker status.

The prevalence of irAEs is thought to be higher with CTLA-4 inhibitors compared to PD-1/PD-L1 inhibitors, and highest with combination therapy. In one study, grade 3-4 irAEs were observed in 16.3%, 27.3% and 55.0% of patients taking nivolumab, ipilimumab, and combined nivolumab plus ipilimumab, respectively [121]. Furthermore, toxicity is thought to be driven more by dose for CTLA-4 inhibitors relative to PD-1/PD-L1 inhibitors [122]. irAEs can occur at any time during treatment, including months after treatment cessation [118,119]. Of note, many studies exclude patients with preexisting autoimmune disease, chronic viral infection, organ transplant, etc. These patients are underrepresented in published research and may respond differently, warranting closer follow-up. Most of the published data on irAEs is not specific to patients treated for UC, and patient demographics may differ.

Despite increased acceptance of ICIs, irAEs remain a significant concern. Ultimately, the benefits of ICI therapy must be weighed against the potential toxicity and detriments to quality of life that irAEs may pose. Further research is needed to identify patients at increased risk for irAEs and to better understand the risks and management protocols that best serve patients.

## Future Directions and Conclusion

Checkpoint blockade has demonstrated safety and efficacy in numerous trials for mUC and high-risk NMIBC leading to multiple FDA approvals. Although recent withdrawn indications for two second-line agents is disappointing, ICIs continue to show clinical efficacy and safety in many settings. These withdrawals affect the United States, but not Europe. Ongoing studies are investigating ICIs in different MIBC settings, including at the neoadjuvant, adjuvant, perioperative, and bladder-sparing stages. In mUC, studies are investigating ICI in combination with novel chemotherapy and immunotherapy agents. One area of need in ICI studies is an analysis of UTUC patients given complexities in staging and subsequent treatment recommendations. An explanation for why minimal data has been published for UTUC patients is that insufficient UTUC enrollment numbers are reached in UC studies to power a UTUC-specific analysis. Future studies should aim to report safety and efficacy data for UTUC patients independently. Given the increased uptake of ICIs, clinicians must be able to recognize irAEs that may accompany these agents. With many ongoing studies incorporating ICIs and novel biomarkers in a variety of pharmacologic and radiotherapeutic regimens, the treatment landscape of UC is evolving rapidly.

## Figures and Tables

**Table 1:** FDA Approved First-line ICIs for mUC.

Study	Phase	Population (Patients)	Intervention(s)	Responses (%)	Survival (months)
KEYNOTE-052	II	370	Pembrolizumab	ORR: 28.6% (95% CI 24.1-33.5%) CR: 8.9% High PD-L1 group ORR: 47.3% (95% CI 37.7-57.0%)	Median OS: 11.3 months (95% CI 9.7-13.1 months) PFS: 2.2 months (95% CI 2.1-3.4 months) High PD-L1 group Median OS: 18.5 months (95% CI 12.2-28.5 months)
KEYNOTE-361	III	1010	Pembrolizumab, Pembrolizumab + cisplatin/carboplatin + gemcitabine	Pending	No statistically significant improvement in OS or PFS in the combined group relative to chemotherapy alone
IMvigor210 (Cohort 1)	II	119	Atezolizumab	Updated ORR: 24% (95% CI 16-32%) Updated CR: 8% High PD-L1 group Original ORR: 28% (95% CI 14%-47%)	Updated Median OS: 16.3 months (95% CI 10.4-24.5 months) Original PFS: 2.7 months (95% CI 2.1-4.2 months)
IMvigor130	III	1213	Atezolizumab, Atezolizumab + cisplatin/carboplatin + gemcitabine	ORR Combination: 47% (95% CI 43-52%) CR: 13% Chemotherapy: 44% (95% CI 39-49%) CR: 7%	Median OS Combination: 16 months (95% CI 13.9-18.9 months) Chemotherapy: 13.4 months (95% CI 12.0-15.2 months) Median PFS Combination: 8.2 months (95% CI 6.5-8.3) Chemotherapy: 6.3 months (95% CI 6.2-7.0)

HR: Hazard Ratio; PFS: Progression Free Survival; ORR: Overall Response Rate; OS: Overall Survival

**Table 2:** Ongoing Studies of First-line Immunotherapy for mUC clinical trial information obtained from ClinicalTrials.gov.

Clinicaltrials.gov Identifier	Intervention(s)	Phase	Estimated Enrollment (Patients)	Estimated Study Date completion	Primary Endpoint
NCT03390595	Avelumab + carboplatin + gemcitabine	II	85	2020	ORR
NCT03324282 (GCISAVE)	Avelumab + cisplatin + gemcitabine	II	90	2022	ORR
NCT03682068 (NILE)	Durvalumab + cisplatin/carboplatin + gemcitabine, Durvalumab + Tremelimumab + cisplatin/carboplatin + gemcitabine	III	885	2022	PFS OS
NCT03459846 (BAYOU)	Durvalumab, Durvalumab + Olaparib	II	154	2021	PFS
NCT03451331	Nivolumab + carboplatin/oxaliplatin + gemcitabine	II	48	2022	ORR
NCT03785925 (PIVOT-10)	Nivolumab + Bempegaldesleukin	II	190	2022	ORR
NCT03036098 (CheckMate901)	Nivolumab + Ipilimumab, Nivolumab + gemcitabine + cisplatin	III	1290	2024	OS PFS
NCT03898180 (LEAP-011)	Pembrolizumab, Pembrolizumab + Lenvatinib	III	694	2022	PFS, OS
NCT03534804 (PemCab)	Pembrolizumab + Cabozantinib	II	39	2023	ORR
NCT03272217	Atezolizumab + Bevacizumab	II	70	2021	OS
NCT03473756	Atezolizumab, Atezolizumab + Rogaratinib	I and II	210	2024	PFS
NCT03093922	Atezolizumab + gemcitabine + cisplatin	II	74	2021	ORR

PFS: Progression Free Survival; ORR: Overall Response Rate; OS: Overall Survival

**Table 3:** FDA Approved Second-line ICIs for mUC.

Study	Phase	Population (Patients)	Intervention(s)	Responses (%)	Survival (months)
IMvigor210 (Cohort 2)	II	310	Atezolizumab	Updated ORR: 16% (95% CI 13-21%) CR: 7% High PD-L1 group Original ORR: 26% (95% CI 18%-36%)	Updated Median OS: 7.9 months (95% CI 6.7-9.3 months) High PD-L1 group Original Median OS: 11.4 months (95% CI 9-NE months)
IMvigor211	III	931	Atezolizumab, docetaxel or paclitaxel or vinflunine	ORR Atezolizumab: 13.4% (10.5-16.9%) Chemotherapy: 13.4% (10.5-16.9%) High PD-L1 group ORR Atezolizumab: 23% (95% CI 15.6-31.9%) Chemotherapy: 21.6% (95% CI 14.5-30.2%)	Median OS Atezolizumab: 8.6 months (95% CI 7.8-9.6 months) Chemotherapy: 8.0 months (95% CI 7.2-8.6 months) High PD-L1 group Median OS Atezolizumab: 11.1 months (95% CI 8.6-15.5 months) Chemotherapy: 10·6 months (95% CI 8.4-12.2 months)
KEYNOTE-045	III	542	Pembrolizumab, docetaxel or paclitaxel or vinflunine	Updated ORR Pembrolizumab: 21.1% Chemotherapy: 11.0%	Updated Median OS Pembrolizumab: 10.1 months Chemotherapy: 7.2 months Updated Median PFS Pembrolizumab: 2.1 months Chemotherapy: 3.3 months
CheckMate275	II	265	Nivolumab	ORR: 19.6% (95% CI 15.0-24.9%)	Median OS: 8.74 months (95% CI 6.05 -NE months
NCT01693562	I/II	191	Durvalumab	ORR: 17.8% (95% CI 12.7%-24.0%)	Median OS: 18.2 months (95% CI 8.1-NE months)
DANUBE	III	1126	Durvalumab, Durvalumab + Tremelimumab	ORR Durvalumab + Tremelimumab: 36% Chemotherapy: 49% High PD-L1 group ORR Durvalumab: 28% Chemotherapy: 48%	Median OS Durvalumab + Tremelimumab: 15.1 months (13.1-18.0 months) Chemotherapy: 12.1 months (10.9-14.0 months) High PD-L1 group Median OS Durvalumab: 14.4 months (10.4-17.3 months) Chemotherapy: 12.1 months (10.4-15.0 months)
NCT01772004 (JAVELIN Solid Tumor)	I	161	Avelumab	ORR: 17% (95% CI 11-24%)	Median OS: 6.5 months (95% CI 4.8-9.5 months)

PFS: Progression Free Survival; ORR: Overall Response Rate; OS: Overall Survival

**Table 4:** Ongoing Studies of Second-line Immunotherapy for mUC clinical trial information obtained from ClinicalTrials.gov.

ClinicalTrials.gov Identifier	Intervention(s)	Phase	Estimated Enrollment (patients)	Estimated Study Date completion	Primary Endpoint
NCT04004442 (COAXIN)	Avelumab + AVB-S6-500	II	31	2022	ORR
NCT03744793	Avelumab + Pemetrexed	II	25	2021	ORR
NCT03891238 (ARIES)	Avelumab	II	67	2021	Efficacy Endpoints OS
NCT04064190	Durvalumab + Vactosertib	II	48	2022	ORR
NCT03871036 (ICRA)	Tremelimumab, Tremelimumab + paclitaxel, Tremelimumab + Durvalumab + paclitaxel	I and II	50	2023	ORR
NCT03824691 (ARCADIA)	Durvalumab + Cabozantinib	II	122	2023	OS
NCT03606174	Nivolumab + Sitravatinib, Pembrolizumab + Sitravatinib + Enfortumab vedotin	II	330	2021	ORR
NCT03980041 (MARIO-275)	Nivolumab, Nivolumab + IPI-549	II	160	2022	ORR
NCT02387996	Nivolmnab	II	386	2021	ORR
NCT03390504	Pembrolizumab, Erdafitinib, docetaxel or vinflunine	III	631	2023	OS
NCT02717156	Pembrolizumab + EphB4-HSA	II	60	2021	OS
NCT02500121	Pembrolizumab	II	108	2020	PFS
NCT03263039 (RESPONDER)	Pembrolizumab	II	80	2020	Biomarkers in clinical responders
NCT02581982	Pembrolizumab + paclitaxel	II	29	2021	ORR
NCT03854474	Pembrolizumab + Tazemetostat	I and II	30	2020	Safety
NCT02500121	Pembrolizumab	II	108	2020	PFS
NCT03737123	Atezolizumab + carboplatin + gemcitabine or Atezolizumab + docetaxel	II	33	2022	PFS
NCT04045613 (FIDES-02)	Derazantinib + Atezolizumab, Derazantinib	I and II	303	2022	ORR
NCT03513952	Atezolizumab + CYT107	II	54	2020	ORR
NCT03237780	Atezolizumab + Eribulin mesylate	II	78	2021	ORR
NCT03179943	Atezolizumab + Guadecitabine	II	53	2022	ORR

PFS: Progression Free Survival; ORR: Overall Response Rate; OS: Overall Survival

**Table 5:** Recent or ongoing phase II and III muscle-invasive urothelial carcinoma clinical trial information obtained from ClinicalTrials.gov.

ClincialTrials.gov Identifier	Intervention(s)	Phase	Estimated Enrollment (patients)	Estimated Study Completion Date	Primary Endpoint	UTUC Included in Enrollment
*Neoadjuvant*						
NCT02736266 (PURE-01)	Pembrolizumab	II	90	2019	pCR	No
NCT03319745	Pembrolizumab	II	20	2020	Toxicity	No
NCT02662309 (ABACUS)	Atezolizumab	II	96	2020	pCR	No
NCT02451423^a^	Atezolizumab	II	42	2021	Change in CD3+ T-cell count, pCR	No
NCT03212651 (PANDORE)	Pembrolizumab	II	41	2019	pCR	No
NCT03294304 (BLASST-1)	Nivolumab, Gemcitabine-Cisplatin	II	43	2020	PaR	No
NCT02690558	Pembrolizumab, Gemcitabine-Cisplatin	II	39	2025	Pathologic downstaging	No
NCT02365766 (HCRN GU14-188)	Pembrolizumab, Gemcitabine-Cisplatin	I/II	83	2021	Safety, PaIR	Yes
NCT03674424 (AURA)	Avelumab, Chemotherapy	II	166	2022	pCR	Yes
NCT04099589	Toripalimab, Gemcitabine-cisplatin	II	60	2022	pCR	Yes
NCT03472274 (DUTRENEO)	Durvalumab, Tremelimumab	II	99	2022	Antitumor activity	Yes
NCT03520491	Nivolumab, Ipilimumab	II	45	2021	Treatment adherence/Toxicity	No
NCT02845323	Nivolumab, Urelumab	II	44	2021	CD8+ T-cell density at cystectomy	No
NCT03549715 (NEMIO)	Durvalumab, Tremelimumab, ddMVAC	I/II	120	2024	Toxicity, pCR	No
NCT03978624	Pembrolizumab, Entinostat	II	20	2022	CD8+ T-cell immune 37-gene signature	No
NCT03832673 (PECULIAR)	Pembrolizumab, Epacadostat	II	38	2020	pCR	No
NCT04289779	Atezolizumab, Cabozantinib	II	42	2023	pCR	No
*Adjuvant*						
NCT02450331 (IMvigor010)	Atezolizumab	III	809	2022	DFS	Yes
NCT03244384 (AMBASSADOR)	Pembrolizumab	III	739	2025	OS, DFS	Yes
NCT02632409 (CheckMate 274)	Nivolumab	III	700	2026	DFS	Yes
NCT03768570	Durvalumab	II	238	2024	DFS	No
*Perioperative*						
NCT03924895 (KEYNOTE-905)	Pembrolizumab	III	610	2026	pCR, EFS	No
NCT03924856 (KEYNOTE-866)	Pembrolizumab, Gemcitabine-Cisplatin	III	790	2025	pCR, EFS	No
NCT03732677 (NIAGARA)	Durvalumab, Gemcitabine-Cisplatin	III	1050	2025	pCR, EFS	No
NCT04209114	Nivolumab, NKTR-214	III	540	2027	pCR, EFS	No
NCT03661320	Nivolumab, Linrodostat, Chemotherapy	III	1200	2026	pCR, EFS	No
*Bladder-Sparing*						
NCT03844256 (CRIMI)	Nivolumab, Ipilimumab, Mitomycin, Capecitabine, Radiotherapy	I/II	50	2023	Toxicity, DLT, DFS	No
NCT03702179 (IMMUNOPRESERVE)	Durvalumab, Tremelimumab, Radiotherapy	II	32	2022	PaR	No
NCT03558087	Nivolumab, Gemcitabine-Cisplatin	II	63	2022	CR	No
NCT04186013	Atezolizumab, Radiotherapy	II	39	2027	pCR	No
NCT04241185 (KEYNOTE-992)	Pembrolizumab, Chemotherapy, Radiotherapy	III	636	2027	Bladder Intact-EFS	No
NCT02662062 (ANZUP 1502)	Pembrolizumab, Cisplatin, Radiotherapy	II	30	2024	Toxicity	No
NCT02621151	Pembrolizumab, Gemcitabine, Radiotherapy	II	54	2026	Bladder Intact-DFS	No
NCT03775265 (SWOG/NRT-1806)	Atezolizumab, Radiotherapy, Gemcitabine or Cisplatin or Fluorouracil, Mitomycin	III	475	2025	Bladder Intact-EFS	No
NCT03617913	Avelumab, Radiotherapy, Fluorouracil, Mitomycin or Cisplatin	II	2	2025	CR	No
NCT03171025 (NEXT)	Nivolmnab, Radiotherapy, Chemotherapy	II	28	2024	FFS	No
NCT03993249	Nivolmnab, Radiotherapy, Chemotherapy	II	78	2021	Locoregional control rate	No

pCR: Pathologic Complete Response; PaIR: Rate of pathologic muscle Invasive Response; PaR: Pathologic Response rate; DSF: Disease Free Survival; OS: Overall Survival; DLT: Dose-limiting Toxicity; EFS: Event-free Survival; CR: Complete Response; FFS: Failure-free Survival

a Enrollment includes NMIBC and MIBC patients. See Table 6 for more detail.

**Table 6:** Recent or ongoing phase II and III non-muscle invasive urothelial carcinoma clinical trial information obtained from ClinicalTrials.gov.

ClincailTrials.gov Identifier	Intervention(s)	Phase	Estimated Enrollment (Patients)	Estimated Study Completion Date	Primary Endpoint
*BCG unresponsive/refractory*					
NCT02625961 (KEYNOTE-057)	Pembrolizumab	II	260	2023	CR, DFS
NCT02844816 (SWOG S1605)	Atezolizumab	II	202	2021	CR, EFS
NCT02451423^a^	Atezolizumab	II	42	2021	Change in CD3+ T-cell count, pCR
NCT02901548	Durvalumab	II	34	2021	CR
NCT03759496	Durvalumab	II	39	2021	MTD, HGRF
NCT03892642 (ABC Trial)	Avelumab, BCG	I/II	27	2025	Proportion of patients receiving a complete induction course
NCT02792192	Atezolizumab, BCG	I/II	24	2021	Safety, DLT (BCG), (BCG), MTD/MAD (BCG), CR
NCT03711032 (KEYNOTE-676)	Pembrolizumab, BCG	III	550	2024	CR
NCT04149574 (CheckMate 7G8)	Nivolmnab, BCG	III	700	2030	EFS
NCT04164082	Pembrolizumab, Gemcitabine	II	163	2023	CR, EFS
NCT03950362 (PREVERT)	Avelumab, Radiotherapy	II	67	2024	High-risk RFS
NCT03317158 (ADAPT-BLADDER)	Durvalumab, BCG or Radiotherapy	I/II	186	2023	Determine the recommended phase 2 dose, RFS
NCT03519256 (CheckMate 9UT)	Nivolmnab, Linrodostat, BCG	II	358	2026	CR, DOCR (CIS participants)
NCT04387461	Pembrolizumab, CG0070	II	37	2022	CR
NCT04106115 (DURANCE)	Durvalumab, S-488210/S-488211	I/II	64	2027	DLT, DFS
*BCG-naïve*					
NCT03799835 (ALBAN)	Atezolizumab, BCG	III	614	2028	RFS
NCT03528694 (POTOMAC)	Durvalumab, BCG	III	975	2024	DFS
NCT03504163	Pembrolizumab	II	37	2021	DFS
*UTUC*					
NCT03345134	Pembrolizumab, BCG	II	20	2021	CR

CR: Complete Response; DFS: Disease Free Survival; EFS: Event-free Survival; pCR: Pathologic Complete Response; MTD: Maximum Tolerated Dose; HGFR: High-grade Relapse Free; DLT: Dose-limiting Toxicity; MAD: Maximum Administered Dose, RFS: Recurrence-free Survival; DOCR: Duration of Complete Response

a Enrollment includes NMIBC and MIBC patients. See Table 5 for more detail.

**Table 7:** Documented toxicities of immune checkpoint inhibitors by organ system obtained from the Clinical Practice Guidelines published by the American Society of Clinical Oncology [120].

Organ System	Documented Toxicities
Skin	• Rash/inflammatory dermatitis • Bullous dermatoses • Steven-Johnson syndrome/toxic epidermal necrolysis • Drug rash with eosinophilia and systemic symptoms
Gastrointestinal	• Colitis • Hepatitis
Lung	• Pneumonitis
Endocrine	• Primary hypothyroidism • Hyperthyroidism • Primary adrenal insufficiency • Hypophysitis • Diabetes
Musculoskeletal	• Inflammatory arthritis • Myositis • Polymyalgia-like syndrome
Renal	• Nephritis
Nervous system	• Myasthenia gravis • Guillain-Barré syndrome • Peripheral neuropathy • Autonomic neuropathy • Aseptic meningitis • Encephalitis • Transverse myelitis
Hematologic	• Autoimmune hemolytic anemia • Acquired thrombotic thrombocytopenic purpura • Hemolytic uremic syndrome • Aplastic anemia • Lymphopenia • Immune thrombocytopenia • Acquired hemophilia
Cardiovascular	• Myocarditis, pericarditis, arrhythmias, impaired ventricular function with heart failure, and vasculitis • Venous thromboembolism
Ocular	• Uveitis/iritis • Episcleritis • Blepharitis

a Documented toxicities are not limited to the examples included in Table 7.
